# Availability of ultrasound imaging of a guidewire parallel to the vein during internal jugular central venous catheter placement

**DOI:** 10.1038/s41598-021-03718-6

**Published:** 2021-12-20

**Authors:** Ryo Wakabayashi

**Affiliations:** Department of Anesthesiology and Resuscitology, Shinshu University School of Medicine, 3-1-1, Asahi, Matsumoto, Nagano 390-8621 Japan

**Keywords:** Health care, Medical research

## Abstract

Ultrasound verification of the guidewire traveling along the vein parallel to it and without any changes in angle has been recommended for prevention of inadvertent arterial catheterization during central venous catheter (CVC) placement. The aim of this study was to determine the availability of this parallel guidewire imaging during internal jugular CVC placement. Fifty-six adult patients undergoing cardiovascular surgery were included. The success rate of acquiring a parallel guidewire image was assessed. Logistic regression models and generalized additive models were used to identify the factors contributing to achieve parallel guidewire imaging. Among 56 patients in whom the guidewire was correctly positioned, the parallel guidewire image was acquired in 45 (80%) patients. Body mass index (crude odds ratio: 0.74 [95% confidence interval: 0.61–0.91]; *p* = 0.004) and distance from the puncture site to the clavicle (crude odds ratio: 1.32 [95% confidence interval: 1.11–1.58]; *p* = 0.002) were associated with successful depiction. The predicted probability of successful visualization was 96% (95% confidence interval: 82–99%) when the distance from the puncture site to the clavicle was 50 mm. The distance is a reliable predictor for successful visualization, and thus it should be considered when performing internal jugular CVC placement.

## Introduction

Inadvertent arterial catheterization during central venous catheter (CVC) placement via the internal jugular vein (IJV) can cause life-threatening complications including hematoma, pseudoaneurysm, arteriovenous fistula, hemothorax, and cerebral infarction^1–3^. Ultrasonography has been recommended to prevent arterial cannulation of a CVC with using for guidance in venous puncture and for identification of guidewire position^4–8^. However, arterial catheterization of a CVC has been reported despite use of ultrasonography^9–13^.

During ultrasound-guided internal jugular CVC placement, inadvertent arterial catheterization has been reported when the guidewire penetration through the posterior wall of the IJV into the adjacent artery was overlooked by simple confirmation of the guidewire within the vein^9–13^. Therefore, not only identification of the guidewire in the IJV but also verification that the guidewire has not penetrated the posterior wall of the IJV is of critical important^7–10,14,15^. Notably, a recent guideline has recommended verification of the guidewire traveling along the IJV parallel to it and without any changes in angle in a long-axis view^8^.

However, the availability of parallel guidewire imaging during internal jugular CVC placement is still unknown. The aims of this study were to determine the availability of parallel guidewire imaging and to identify the factors contributing to successful visualization of the parallel guidewire during internal jugular CVC placement in adult patients.

## Methods

This single-center, prospective, observational study was approved by the Committee for Medical Ethics of Shinshu University School of Medicine (Matsumoto, Nagano, Japan; approval no. 3580; December 8, 2016). Written informed consent was obtained from all patients before enrollment. All methods were carried out in accordance with relevant guidelines and regulations.

This study enrolled patients aged 20 years or older who were scheduled to undergo cardiovascular surgery under general anesthesia and who required central venous catheterization via the right IJV and intraoperative transesophageal echocardiography (TEE) monitoring. Exclusion criteria included a history of neck surgery, cervical vertebra disease or cervical myelopathy, subcutaneous abscess or hematoma of the neck, anatomical abnormality of blood vessels in the neck or chest, preexisting heart rhythm device leads, and contraindications to TEE such as perforated viscus or esophageal stricture.

In the operating room, standard monitoring (pulse oximetry, electrocardiogram, and noninvasive blood pressure) was carried out during the procedure. General anesthesia was induced with 1–2 mg/kg propofol and 0.1–0.2 μg/kg/min remifentanil. After 0.6–0.8 mg/kg rocuronium had been intravenously administered, the trachea was intubated. The lungs were mechanically ventilated with a tidal volume of 6–8 mL/kg of predicted body weight and a positive end-expiratory pressure of 5 cm of water. Anesthesia was maintained with 1.7–2.0% sevoflurane in 45% oxygen and 0.05–0.1 μg/kg/min remifentanil. After induction of general anesthesia, an X7-2t probe (Philips Healthcare, Bothell, WA, USA) was inserted and an iE33 ultrasound machine (Philips Healthcare, Bothell, WA, USA) was used for TEE monitoring. The TEE probe was positioned at the mid-esophagus and a bicaval view was obtained to visualize the superior vena cava and right atrium. Each patient was placed in a 15-degree Trendelenburg position with the patient's head rotated 30 degrees to the left according to the guidelines for central venous access^4–8^.

**Figure 1:**
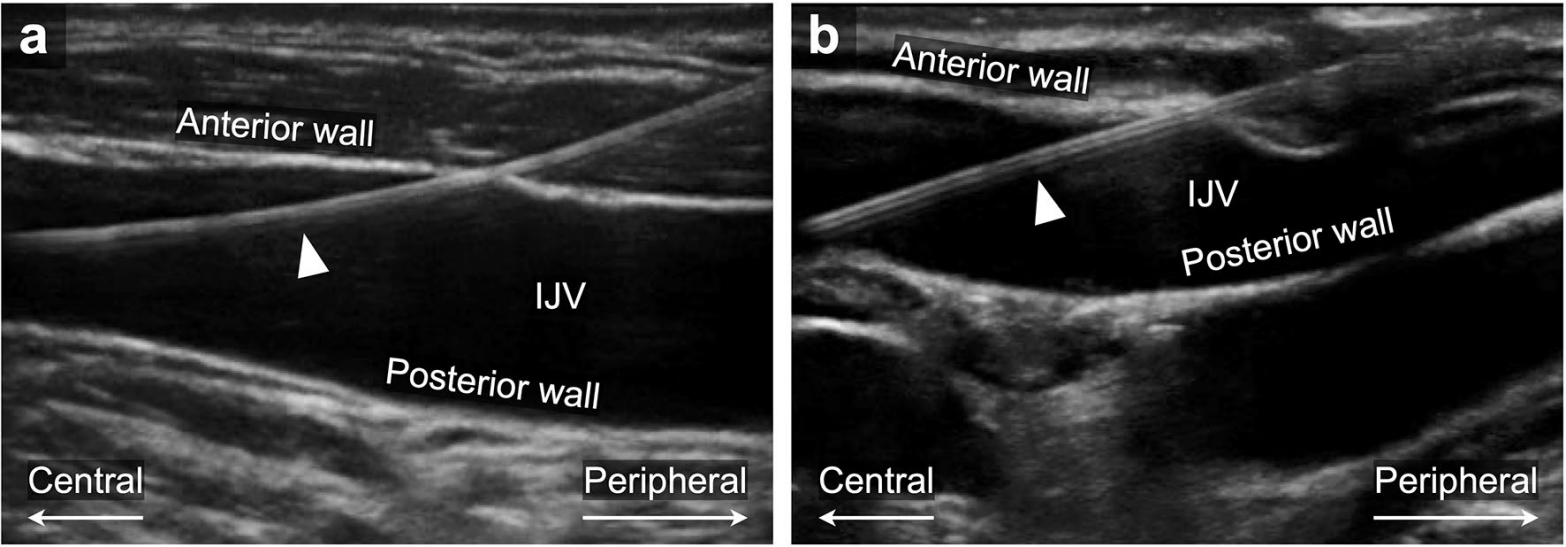
(**a**) Parallel guidewire image. The guidewire is traveling along the IJV parallel to it and without any changes in angle (arrowhead). (**b**) Non-parallel guidewire image. The guidewire is not traveling along the IJV parallel to it (arrowhead). IJV internal jugular vein.

Ultrasound-guided CVC placement was performed by two cardiac anesthesiologists with more than 5 years of experience who were familiar with ultrasound devices. An S-Nerve ultrasound device (SonoSite, Bothell, WA, USA) with a 13–6 MHz 38-mm linear array transducer was used for ultrasonography. Before the procedure, the depth and sagittal diameter of the right IJV at the height of the apex of the triangle delimited by the two heads of the right sternocleidomastoid muscle and the right clavicle. Real-time ultrasound guidance was used for the puncture of a 20-gauge catheter over the needle, with an effective puncture length of 34 mm, into the right IJV in a short-axis view according to the guidelines for central venous access^4–8^. The puncture was performed at the height of the apex of the triangle delimited by the two heads of the right sternocleidomastoid muscle and the right clavicle (the apex of Sedillot's triangle) with an angle of 45° to the skin according to the standard method recommended^8,16^. After obtaining blood backflow, the needle was removed while leaving the plastic catheter in place and intravenous placement of the catheter was confirmed by pressure measurement. A 0.015-in. guidewire was then inserted with a length of 15–20 cm from the skin puncture site, and the guidewire passing in the right IJV was dynamically depicted by ultrasonography in a long-axis view with the transducer positioned to touch the right clavicle. Whether the guidewire is traveling along the IJV parallel to it and without any changes in angle (Fig. 1a) or not (Fig. 1b) was assessed offline by a blinded investigator who was familiar with ultrasonography and CVC placement. TEE was used for final confirmation of correct guidewire placement^17^. After placement of a CV RegaForce EX LG-EX122TC (Terumo Co., Ltd., Tokyo, Japan)^18^ and removal of the guidewire, the CVC position was confirmed by a chest X-ray and the distance from the puncture site to the right clavicle was measured.

The primary outcome was the success rate of acquiring a parallel guidewire image. The secondary outcomes were the factors contributing to successful visualization of the parallel guidewire.

Binary and categorical variables were reported as numbers (proportions) and were compared using the chi-square test or Fisher's exact test. Normally distributed continuous variables were expressed as means (standard deviation) and were compared using the *t*-test. Non-normally distributed continuous variables were expressed as medians (interquartile) and were compared using the Mann–Whitney *U* test. Logistic regression analysis was conducted to identify the predicting factors and to estimate the odds ratios (ORs) with 95% confidence intervals (CIs) for successful visualization of the parallel guidewire. The variables including body mass index (BMI), depth of the IJV, sagittal diameter of the IJV, and distance from the skin puncture site to the clavicle were used for the regression models according to a previous study^14^. Correlation analysis was performed to elucidate the correlations between variables used in logistic analysis. Emphasizing clinical relevance, generalized additive models with a locally weighted scatterplot smoother were used to calculate the predicted probability of successful depiction of the parallel guidewire image and to draw spline curves indicating the relationships of successful visualization of the parallel guidewire image with those variables. The sample size was not explicitly determined to provide sufficient power for performing statistical analyses because this study was designed as an exploratory study. All analyses were performed using SAS University Edition (SAS Institute, Cary, NC, USA). A *p* value of less than 0.05 was considered statistically significant in all statistical tests.

## Results

**Figure 2:**
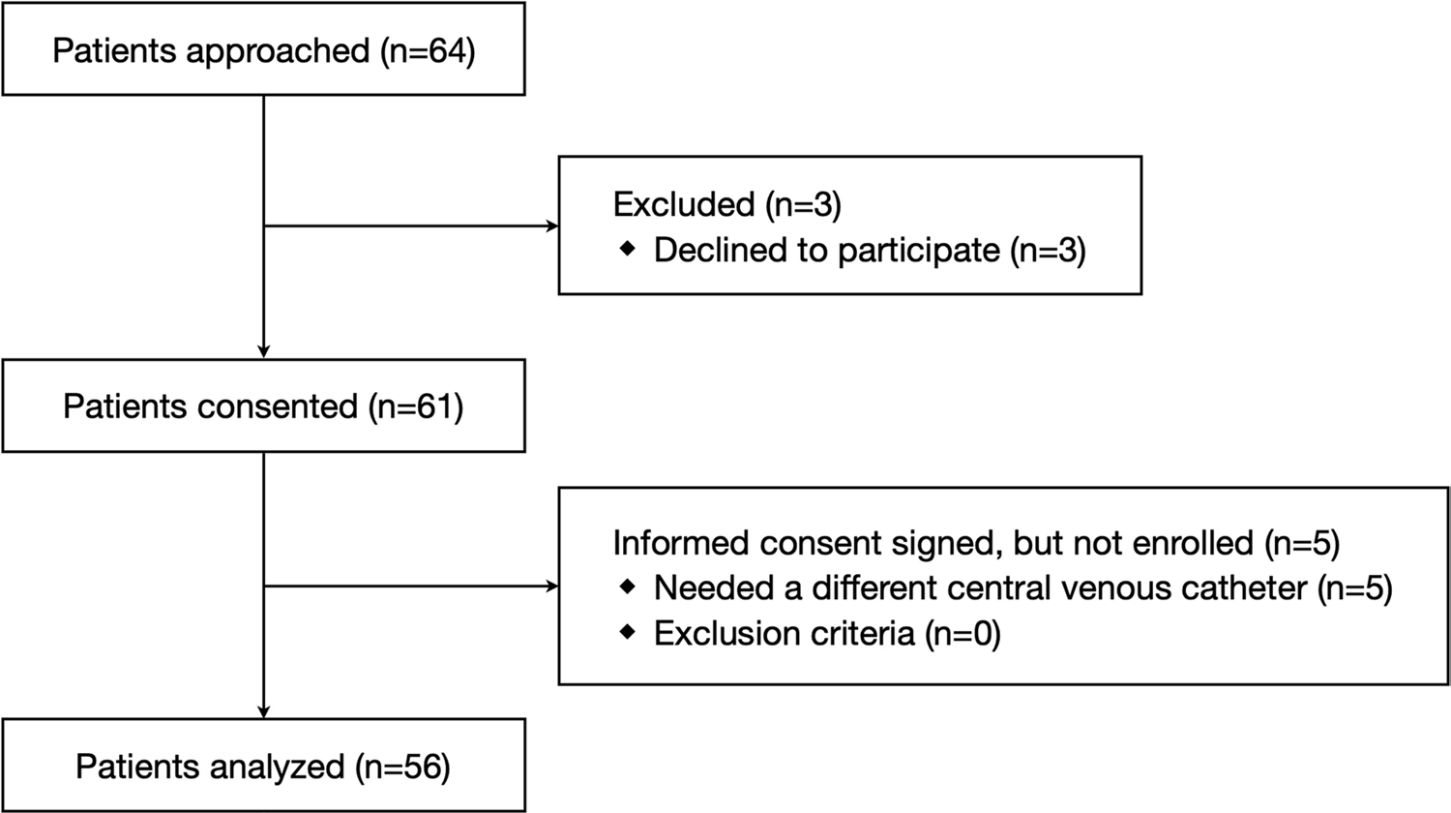
Patients flow diagram.

From December 2016 to March 2019, the eligibility of 64 patients was assessed and 8 patients were excluded. In total, 56 patients were included in analyses (Fig. 2). In all of the patients, correct guidewire placement was verified by TEE and correct CVC placement was achieved. The characteristics of the patients are shown in Table 1. Among the 56 patients included in this study, the parallel guidewire image was acquired in 45 (80% [95% CI 70–91%]) patients (success group) and was not acquired in 11 patients (failure group). The patients in the success group showed significantly lower weight, lower BMI, and longer distance from the puncture site to the clavicle than did the patients in the failure group (Table 1).

**Table 1:** Comparison of the characteristics of patients in whom parallel guidewire imaging succeeded or failed.

	All patients (*n* = 56)	Parallel guidewire imaging		*p* value
		Success (*n* = 45)	Failure (*n* = 11)	
Age (years)	75 (67–81)	75 (68–80)	71 (66–82)	0.96
Male sex	37 (66%)	20 (67%)	17 (65%)	0.85
Height (cm)	157 (154–164)	157 (154–164)	162 (153–169)	0.94
Weight (kg)	57 (51–67)	56 (48–64)	61 (54–82)	0.02
Body mass index (kg/m^2^)	22.5 (20.2–24.8)	21.9 (20.0–24.1)	27.3 (21.6–32.6)	0.004
Depth of the IJV (mm)	8 (6–9)	8 (6–9)	7 (6–10)	0.80
Sagittal diameter of the IJV (mm)	10 (8–13)	10 (8–13)	11 (8–14)	0.39
Distance from the puncture site to the clavicle (mm)	48 (42–54)	48 (44–56)	39 (33–42)	< 0.001

Data are presented as median (interquartile) or number (proportion). *IJV* internal jugular vein.

The results of logistic regression analysis are shown in Table 2. There were significant associations of successful depiction of the parallel guidewire with BMI (crude OR: 0.74 [95% CI 0.61–0.91]; *p* = 0.004, AUC: 0.77 [95% CI 0.64–0.91]) and distance from the puncture site to the clavicle (crude OR: 1.32 [95% CI 1.11–1.58]; *p* = 0.002, AUC: 0.87 [95% CI 0.77–0.97]). In correlation analysis, there was a positive correlation between BMI and depth of the IJV in all of the patients (*r* = 0.38; *p* = 0.004).

**Table 2:** Univariate logistic regression for parallel guidewire imaging.

Variables	Intercept (SE)	Coefficient (SE)	OR (95% CI)	*p* value
Body mass index (kg/m^2^)	8.61 (2.59)	− 0.30 (0.10)	0.74 (0.61–0.91)	0.004
Depth of the IJV (mm)	2.00 (1.05)	− 0.08 (0.13)	0.93 (0.72–1.19)	0.54
Sagittal diameter of the IJV (mm)	2.95 (1.27)	− 0.14 (0.11)	0.87 (0.71–1.07)	0.19
Distance from the puncture site to the clavicle (mm)	− 10.85 (3.74)	0.28 (0.09)	1.32 (1.11–1.58)	0.002

*CI* confidence interval; *IJV* internal jugular vein; *OR* odds ratio; *SE* standard error.

**Figure 3:**
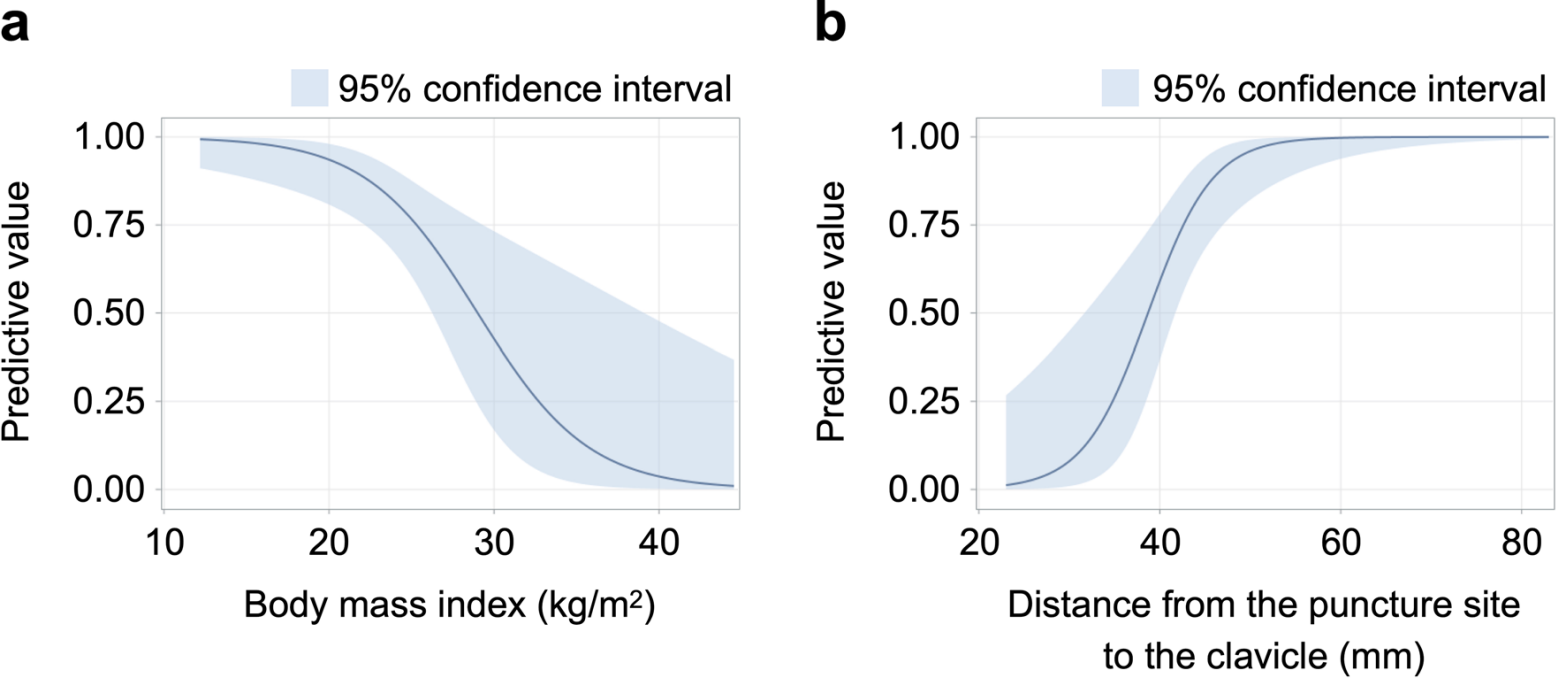
Predicted probability for successful visualization of the parallel guidewire from general additive model. (**a**) Relationship between body mass index and probability of successful visualization of the parallel guidewire. (**b**) Relationship between distance from the puncture site to the clavicle and probability of successful visualization of the parallel guidewire. Trend lines are presented as dark blue lines and the blue ranges indicate 95% confidence intervals.

The predicted probability of successful depiction of the parallel guidewire from general additive models is shown in Fig. 3. The predicted probability of successful visualization decreased with increase in BMI and increased with increase in distance from the puncture site to the clavicle. The predicted probabilities of successful visualization based on the representative values of BMI and distance from the puncture site to the clavicle are shown in Table 3. The predicted probability of successful visualization was 96% (95% CI 82–99%) when the distance from the puncture site to the clavicle was 50 mm.

**Table 3:** Predicted probabilities for successful visualization of parallel guidewire image based on representative values of body mass index and distance from the puncture site to the clavicle.

	Predicted probability	95% CI
**Body mass index (kg/m**^**2**^**)**		
20	93	81–98
30	42	17–73
40	4	0–48
**Distance from the puncture site to the clavicle (mm)**		
40	59	37–78
50	96	82–99
60	100	94–100

*CI* confidence interval.

## Discussion

The present study showed that the success rate of acquiring a parallel guidewire image for prevention of inadvertent arterial catheterization during internal jugular CVC placement was 80% when the guidewire was correctly positioned. This study also showed that BMI and distance from the puncture site to the clavicle were associated with successful visualization of the parallel guidewire.

It has been reported that puncture of the posterior wall of the IJV during ultrasound-guided CVC placement occurs in 34–64% of the procedures^19,20^. Thus, final confirmation in ultrasound images that the guidewire has not penetrated the posterior wall of the vein before dilator insertion has an important role for preventing arterial catheterization and consequent life-threatening complications^8^. However, inadvertent arterial catheterization during CVC placement has been reported in many previous cases in which venous residence of a guidewire was confirmed in a short-axis view^11,12^. Furthermore, in a recently reported case, inadvertent arterial catheterization occurred despite verifying the guidewire within the vein in both a short-axis view and a long-axis view^10^. Therefore, confirmation of the guidewire gradually coming into contact with the posterior wall of the IJV in a long-axis view is very important^8^. In the current study, the parallel guidewire image was obtained only in 80% of the patients. This result suggests that availability of parallel guidewire imaging is insufficient and additional procedures for confirmation are required in some patients.

The results of logistic regression analysis suggest that low BMI and long distance from the puncture site to the clavicle contribute to successful visualization of the parallel guidewire. Importantly, the logistic model using distance from the puncture site to the clavicle showed an AUC value of 0.87, indicating good accuracy for predicting successful depiction of the parallel guidewire. In the current study, a standard puncture height of the apex of the triangle delimited by the two heads of the sternocleidomastoid muscle and the clavicle^8^ was used. Thus, the results imply that the parallel guidewire is difficult to visualize in patients who have a short neck. Kayashima et al. showed that a shorter distance from the puncture site to the clavicle is associated with a shorter length of the guidewire that is visible in the IJV in a long-axis view^14^. Therefore, difficulty in depicting the parallel guidewire in patients who have a short neck could be explained by the small region of interest to assess whether the guidewire path is parallel to the vein due to short length of the guidewire observed within the IJV.

The results also suggest that a high BMI disturbs successful visualization of the parallel guidewire. As shown in this study, obese patients have a deep IJV^21^. Additionally, increased BMI is associated with a large cross-sectional area of the IJV, especially at lower cervical levels^22,23^. Although the results did not reveal associations of successful visualization of the parallel guidewire with depth and sagittal diameter of the IJV, a deep and large IJV might lead to caudal venipuncture and a linear guidewire path that is observed within the IJV, possibly resulting in failed depiction of the parallel guidewire. High BMI is also accompanied by a short neck^21^. However, there was no significant correlation between BMI and distance from the puncture site to the clavicle in this study.

The results obtained by using the generalized additive models suggest that a clinically important cut-off value of the distance from the puncture site to the clavicle is 50 mm or more, indicating a predicted probability for successful depiction of the parallel guidewire of 96%. Therefore, in patients with the distance between the apex of Sedillot's triangle and the clavicle being less than 50 mm, depiction of the parallel guidewire may not be a useful method for confirming safe CVC placement. In such patients, if an operator uses a short needle with an effective puncture length being within 50 mm, the operator can determine whether the guidewire is placed in the jugular vein or not by observing area within which the needle reached. In that situation, there is no need to observe more than 50 mm. In other words, even if the guidewire is not placed parallel to the posterior wall, the operator can confirm whether the guidewire is safely inserted or not. The data obtained in this study indicate that a short needle should be safer than a long needle during ultrasound-guided internal jugular central venous catheterization. Also, although puncture at 50 mm or more cephalad from the clavicle might result in successful depiction of the parallel guidewire, puncture without considering the sternocleidomastoid muscle may cause problems such as difficulty in inserting the dilator and/or postoperative patient discomfort.

This study had several limitations. First, application of the results to other institutions may be limited because this was a single-center study. Second, a 38 mm linear array transducer was used for verification of the parallel guidewire image. Whether the parallel guidewire image can be obtained or not possibly depends on the width of the ultrasound transducer. A transducer narrower than 38 mm would provide a better view and higher rate of successful depiction of the parallel guidewire than the transducer used in this study. Finally, the quality of the guidewire including stiffness and ultrasound visibility depends on the manufacturer. Use of other types of guidewire might lead to different results.

In conclusion, a parallel guidewire image for prevention of inadvertent arterial catheterization was obtained only in 80% of adult patients during internal jugular CVC placement. Low body mass index and long distance from the puncture site to the clavicle were associated with successful visualization of the parallel guidewire. Importantly, distance from the puncture site to the clavicle was a reliable predictor for successful depiction of the parallel guidewire, and the distance of 50 mm indicated a predicted probability for successful visualization of 96%.

## Data Availability

The datasets generated during and/or analyzed during the current study are not publicly available due to concerns about backtracking of personal information of study subjects but are available from the corresponding author on reasonable request.
